# The Role of TNF-α in Ischemic Stroke

**DOI:** 10.3390/ijms27031424

**Published:** 2026-01-30

**Authors:** Renata Kołodziejska, Hanna Pawluk, Agnieszka Tafelska-Kaczmarek, Mateusz Pawluk, Krzysztof Koper, Antoni Godlewski, Julia Kuk, Krzysztof Sergot, Natalia Kurhaluk, Alina Woźniak

**Affiliations:** 1 Department of Medical Biology and Biochemistry, Faculty of Medicine, Collegium Medicum in Bydgoszcz, Nicolaus Copernicus University in Toruń, Karłowicza 24, 85-092 Bydgoszcz, Poland; hannapawluk1@wp.pl (H.P.); pawluk.mateusz23@gmail.com (M.P.); alina-wozniak@wp.pl (A.W.); 2 Department of Organic Chemistry, Faculty of Chemistry, Nicolaus Copernicus University in Toruń, Gagarina 7, 87-100 Toruń, Poland; tafel@umk.pl; 3 Department of Surgical Oncology, Oncology Center, Faculty of Health Sciences, Collegium Medicum in Bydgoszcz, Nicolaus Copernicus University in Toruń, Romanowskiej Street 2, 85-796 Bydgoszcz, Poland; krzysztof.koper@cm.umk.pl; 4 Student Scientific Club of Biochemistry and Bioorganic Chemistry, Department of Medical Biology and Biochemistry, Faculty of Medicine, Collegium Medicum in Bydgoszcz, Nicolaus Copernicus University in Toruń, Karłowicza 24, 85-092 Bydgoszcz, Poland; 319905@stud.umk.pl (A.G.); 319910@stud.umk.pl (J.K.); 5 Laboratory of Laser Molecular Spectroscopy, Institute of Applied Radiation Chemistry, Faculty of Chemistry, Lodz University of Technology, Wroblewskiego 15, 93-590 Lodz, Poland; krzysztofsergot@gmail.com; 6 Institute of Biology, Pomeranian University in Słupsk, 76-200 Słupsk, Poland; natalia.kurhaluk@upsl.edu.pl

**Keywords:** TNF-α, ischemic stroke, pathomechanism, pharmacological strategies

## Abstract

Ischemic stroke accounts for approximately 80–85% of all stroke cases and triggers a complex cascade of metabolic, immunological, and neurodegenerative processes. Among the key mediators involved, TNF-α occupies a central position due to its distinctly dual and phase-dependent actions. Importantly, the biological effects of TNF-α are not static but evolve dynamically over time following ischemic insult. During the acute phase of ischemia, a rapid increase in TNF-α levels, primarily originating from activated microglia, leads to the predominant activation of the TNFR1 receptor. This results in enhanced apoptosis and necroptosis, disruption of the blood–brain barrier, increased leukocyte recruitment, and the progression of secondary neuronal injury. In later phases, the role of TNF-α shifts, with signaling through TNFR2 becoming more prominent, thereby supporting reparative mechanisms, including neurogenesis, angiogenesis, and synaptic remodeling. The dual nature of TNF-α means that both its excessive activation and complete inhibition may produce detrimental effects. Notably, the therapeutic relevance of TNF-α critically depends on the timing of intervention relative to stroke onset. A comprehensive analysis of current evidence underscores the central, temporally and contextually dependent role of TNF-α in the pathophysiology of ischemic stroke. It also indicates that future therapeutic strategies should aim to selectively suppress the harmful TNFR1-mediated signaling while preserving or enhancing TNFR2-dependent neuroprotective pathways. Such time-sensitive and receptor-selective modulation holds promise for limiting acute ischemic injury and promoting endogenous repair processes, representing a compelling direction for the development of next-generation neuroprotective therapies.

## 1. Introduction

Ischemic stroke is the most common type of stroke, accounting for approximately 80–85% of all cases. Its epidemiology indicates a steadily increasing population burden, driven both by societal aging and by the rising prevalence of major risk factors such as hypertension, atherosclerosis, diabetes, and metabolic disorders [1,2,3]. Despite advances in acute-phase therapy, including thrombolysis and mechanical thrombectomy, ischemic stroke remains one of the leading causes of death and long-term disability worldwide [4]. Its complex pathophysiology involves dynamic interactions between ischemia, inflammatory processes, and blood–brain barrier (BBB) disruption, making immunological mediators key regulators of disease progression [5,6].

Against this background, evaluating the role of tumor necrosis factor alpha (TNF-α) in the pathogenesis, diagnosis, and potential therapeutic strategies for ischemic stroke becomes particularly important, as this cytokine lies at the center of the inflammatory axis activated following an ischemic episode. After an acute drop in cerebral perfusion, TNF-α is among the most rapidly induced pro-inflammatory mediators, as demonstrated in both animal models and patients with acute stroke [7,8,9]. Its expression rises sharply in activated microglia and infiltrating monocytes, initiating a cascade of pro-inflammatory responses that includes nuclear factor kappa-light-chain-enhancer of activated B cells (NF-κB) activation, reactive oxygen species (ROS) production, and the induction of adhesion molecules, all of which contribute to the progression of neuroinflammatory tissue damage [10,11].

An increasing body of evidence suggests that TNF-α’s effects in ischemic stroke are time-dependent and depend on selective receptor activation. In the acute phase of ischemia, signaling mediated by tumor necrosis factor receptor 1 (TNFR1; TNFRSF1A, CD120a, p55) predominates. Through its death domain, TNFR1 activates pro-inflammatory pathways and programmed cell death, contributing to secondary neuronal injury and the disruption of the blood–brain barrier [8,9].

In the subacute and chronic phases, activation of tumor necrosis factor receptor 2 (TNFR2; TNFRSF1B, CD120b, p75) becomes more prominent. Lacking a death domain, TNFR2 initiates pro-survival and reparative signaling pathways, including non-canonical NF-κB activation, thereby supporting neuroprotection, angiogenesis, and regenerative processes. In this context, TNF-α functions as a mediator of neural tissue repair and adaptation [8,9].

From a diagnostic perspective, elevated TNF-α concentrations in blood and cerebrospinal fluid correlate with the extent of ischemic injury, the intensity of the inflammatory response, and unfavorable clinical outcomes, particularly in the acute phase of stroke. These associations highlight its potential utility as a biomarker of early inflammatory processes [12,13,14]. From a therapeutic perspective, there is an increasing emphasis on precise modulation rather than global inhibition of TNF-α signaling. Strategies that selectively suppress TNFR1-dependent pathways while preserving or enhancing the TNF-α–TNFR2 axis may help limit acute brain injury without disrupting reparative and regenerative mechanisms [15].

The multidimensional, dynamic, and context-dependent role of TNF-α suggests that considering both temporal factors and receptor selectivity may be essential for developing more effective and personalized diagnostic and therapeutic strategies for ischemic stroke.

## 2. TNF-α Biology: Sources, Receptors, Signaling Pathways

TNF-α is the prototypical member of the type II transmembrane TNF superfamily, which comprises 30 receptors and 19 related ligands with diverse functions in cell differentiation, inflammation, immunity, and apoptosis [16,17,18]. TNF-α exhibits stimulus-dependent secretion dynamics and substantial functional plasticity in various pathological conditions, such as bacterial infections and systemic inflammatory responses [8,19]. It plays an important role in mammalian immunity and cellular homeostasis. The main sources of this cytokine are monocytes, macrophages, T lymphocytes, and mast cells [16]. Its expression is tightly regulated by pathogenic and endogenous stimuli, including lipopolysaccharide (LPS), interleukin 1 (IL-1), and interleukin 6 (IL-6). LPS-induced TNF production critically requires the activation of mammalian uncoordinated-13 homolog 4 (Munc13-4), a protein indispensable for vesicle priming and cytokine secretion. At the transcriptional level, NF-κB plays a pivotal role, rapidly translocating to the nucleus upon activation and binding to enhancer elements within the TNF gene promoter. Its expression is additionally modulated by activator protein-1 (AP-1) and CCAAT/enhancer-binding protein (C/EBP), which together form a multilayered, tightly controlled regulatory network [20].

TNF signaling is mediated by two isoforms: the transmembrane form (mTNF) and the soluble form (sTNF). The transmembrane form, which serves as the biosynthetic precursor of sTNF-α, can be proteolytically processed by a disintegrin and metalloproteinase domain-containing protein 17 (ADAM17), also known as TNF-α converting enzyme (TACE), thereby releasing the soluble isoform [21,22]. Both forms of TNF interact with two specialized membrane receptors: tumor necrosis factor receptor 1 and tumor necrosis factor receptor 2 [23,24].

Both receptors contain four homologous cysteine-rich domains in their extracellular regions; however, their intracellular regions differ substantially in structure and function, which ultimately determines their distinct physiological role [25]. TNFR1, which is ubiquitously expressed on most cell types, contains a death domain (DD) that enables interactions with other signaling proteins. It mediates the cytotoxic, pro-inflammatory, and proapoptotic effects of TNF and can be activated by both mTNF and sTNF [25,26]. In contrast, TNFR2 lacks a death domain but contains an intracellular region that facilitates binding to TRAF proteins. TNFR2 is expressed predominantly on immune cells, endothelial cells, and glial cells, and plays a role in T-cell activation and proliferation, as well as angiogenesis. Although TNFR2 can bind both mTNF and sTNF, its full signaling activation is achieved only in response to mTNF [16,17,21,25,26,27,28,29].

### 2.1. TNFR1 Signaling

**Figure 1:**
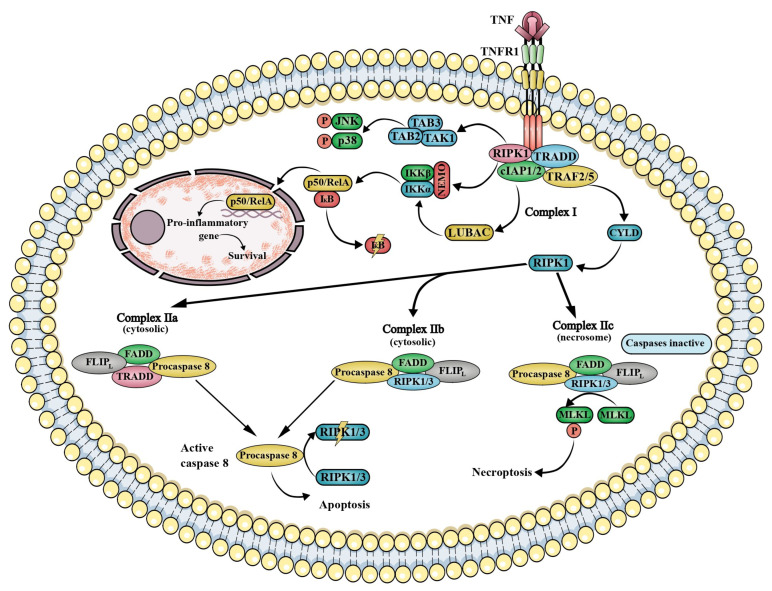
TNFR1 Signaling. Tumor necrosis factor receptor (TNFR) signaling can lead to cell survival or cell death, depending on the composition and dynamics of the signaling complexes formed. Four major complexes are distinguished: Complex I, Complexes IIa and IIb, and Complex IIc (the necrosome), each mediating distinct cellular outcomes. Complex I assembles in the presence of ubiquitinated RIPK1 and triggers activation of NF-κB, JNK, and p38 MAPK pathways, resulting in the induction of pro-inflammatory mediators and the initiation of survival signaling. In contrast, Complexes IIa and IIb form under conditions where RIPK1 is not ubiquitinated and caspases are active, leading to caspase-8 activation and the execution of apoptosis. Finally, Complex IIc, known as the necrosome, is generated in the presence of non-ubiquitinated RIPK1 and inactive caspases, thereby initiating necroptosis, a regulated form of necrotic cell death. Abbreviations: cIAP1/2—cellular inhibitor of apoptosis protein 1 and 2; CYLD—CYLD lysine 63 deubiquitinase; FADD—Fas-associated death domain protein; FLIP_L_—FLICE-like inhibitory protein, long isoform; IKKα—inhibitor of nuclear factor κB kinase subunit alpha; IKKβ—inhibitor of nuclear factor κB kinase subunit beta; IκB—inhibitor of κB; JNK—c-Jun N-terminal kinase; LUBAC—linear ubiquitin chain assembly complex; MLKL—mixed lineage kinase domain-like protein; NEMO—NF-κB essential modulator (also known as IKKγ); p38—p38 mitogen-activated protein kinase (p38 MAPK); p50—NF-κB subunit p50; RelA—RELA proto-oncogene, NF-κB subunit p65; RIPK1/3—receptor-interacting serine/threonine-protein kinase 1/3; TAB2—TAK1-binding protein 2; TAB3—TAK1-binding protein 3; TAK1—transforming growth factor beta-activated kinase 1; TNF—tumor necrosis factor; TNFR1—tumor necrosis factor receptor 1; TRADD—TNF receptor type 1–associated death domain protein, TRAF2/5—TNF receptor–associated factors 2 and 5.

Binding of TNF to TNFR1 induces receptor trimerization and triggers the sequential assembly of distinct intracellular signaling complexes, namely membrane-associated complex I and cytosolic complexes IIa, IIb, and IIc (Figure 1) [30].

Complex I assembly is initiated by recruitment of TNF receptor–associated death domain protein (TRADD), receptor-interacting protein kinase 1 (RIPK1), and TNF receptor–associated factors 2 and 5 (TRAF2/5), which facilitate the recruitment of cellular inhibitor of apoptosis proteins 1 and 2 (cIAP1/2) and linear ubiquitin chain assembly complex (LUBAC)-mediated RIPK1 ubiquitination [17,30,31,32]. Ubiquitinated receptor-interacting protein kinase 1 activates the canonical NF-κB pathway via the IκB kinase (IKK) complex, composed of inhibitor of nuclear factor κB kinase subunit alpha (IKKα), inhibitor of nuclear factor κB kinase subunit beta (IKKβ), and the regulatory subunit nuclear factor κB essential modulator (NEMO/IKKγ), resulting in phosphorylation and proteasomal degradation of inhibitor of nuclear factor κB alpha (IκBα) and subsequent nuclear translocation of NF-κB dimers, predominantly p50/RelA (p65) [33,34,35]. Concurrently, mitogen-activated protein kinase kinase kinase 7 (MAP3K7), also known as transforming growth factor beta–activated kinase 1 (TAK1), in complex with TAK1-binding proteins 1 and 2 (TAB1/2), phosphorylates IKKβ and activates mitogen-activated protein kinase cascades, resulting in c-Jun N-terminal kinase (JNK) and p38 mitogen-activated protein kinase (p38 MAPK) activation. Coordinated NF-κB and MAPK signaling downstream of complex I promotes inflammatory gene expression and cell survival, supporting effective immune responses [36].

In contrast, insufficient RIPK1 ubiquitination redirects tumor necrosis factor receptor 1 signaling toward cytosolic death-inducing complexes. Non-ubiquitinated RIPK1 promotes formation of complex IIa, comprising TRADD, Fas-associated death domain protein (FADD), procaspase-8, and FLICE-like inhibitory protein long isoform (FLIPL), or complex IIb, which lacks TRADD and forms upon cIAP1/2 depletion [30,37,38]. Both complexes activate caspase-8 and induce apoptosis. When caspase-8 activity is impaired, complex IIc forms, leading to necroptosis through the assembly of the receptor-interacting protein kinase 1 and 3 (RIPK1–RIPK3) necrosome and subsequent activation of the mixed lineage kinase domain-like pseudokinase (MLKL), which oligomerizes and disrupts plasma membrane integrity [30,39,40].

### 2.2. TNFR2 Signaling

**Figure 2:**
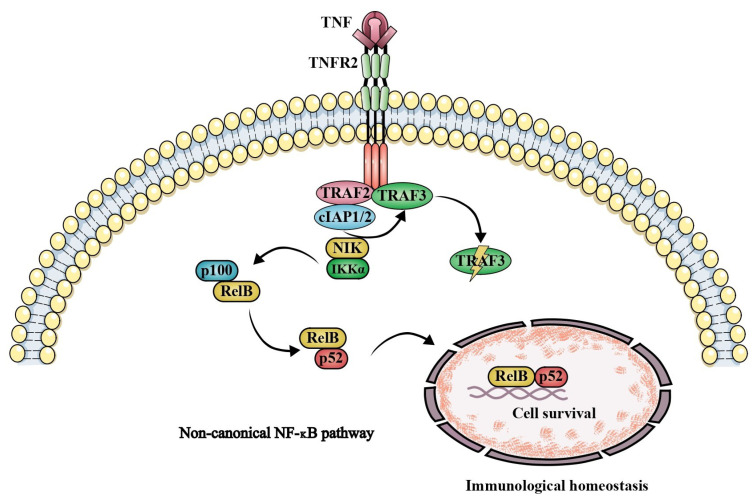
TNFR2-mediated activation of the noncanonical NF-κB signaling pathway. The engagement of tumor necrosis factor receptor 2 (TNFR2) initiates a signaling cascade that converges on the noncanonical NF-κB pathway, a pathway shared by selected members of the TNF receptor superfamily. This pathway is critically dependent on NF-κB–inducing kinase (NIK), which drives phosphorylation-dependent processing of the p100 precursor into p52. The resulting p52/RelB heterodimers translocate to the nucleus, where they regulate gene expression associated with cell survival, differentiation, and tissue repair. Abbreviations: cIAP1/2—cellular inhibitor of apoptosis protein 1 and 2; IKKα—inhibitor of nuclear factor κB kinase subunit alpha; NF-κB—nuclear factor kappa-light-chain-enhancer of activated B cells; NIK—NF-κB-inducing kinase; p52—NF-κB subunit p52; p100—NF-κB subunit p100; RelB—v-rel avian reticuloendotheliosis viral oncogene homolog B; TNF—tumor necrosis factor; TNFR1—tumor necrosis factor receptor 2; TRAF2/3—TNF receptor-associated factor 2 and 3.

Tumor necrosis factor receptor 2 regulates cell differentiation, survival, immunoregulation, and angiogenesis and activates both canonical and noncanonical NF-κB signaling pathways (Figure 2) [41]. While canonical NF-κB activation is rapid and typically initiated by TNFR1, leading to induction of nuclear factor of kappa light polypeptide gene enhancer in B-cells inhibitor delta (p100), noncanonical NF-κB signaling is slower, sustained, and driven by selected tumor necrosis factor receptor superfamily members, including TNFR2 [30,42].

TNFR2-mediated noncanonical NF-κB signaling depends on nuclear factor kappa B-inducing kinase (NIK), which, under basal conditions, is restrained by tumor necrosis factor receptor–associated factors 2 and 3, as well as cellular inhibitor of apoptosis proteins 1 and 2 (cIAP1/2). Upon activation, NIK phosphorylates IκB kinase alpha, triggering processing of p100 to p52 and formation of the transcriptionally active p52/RelB complex that controls immune differentiation, cell survival, and immune homeostasis (Figure 2) [42,43,44].

Although TNFR2 lacks a death domain, TRAF2 degradation links TNFR2 signaling to cell death pathways by impairing canonical NF-κB activation and facilitating the formation of cytosolic complexes IIa, IIb, and IIc, thereby promoting apoptosis or necroptosis. Thus, the biological outcome of tumor necrosis factor signaling reflects the balance between tumor necrosis factor receptor 1 and tumor necrosis factor receptor 2, which are regulated by distinct signaling pathways [30,45].

## 3. The Role of TNF-α in the Pathomechanism of Stroke

**Figure 3:**
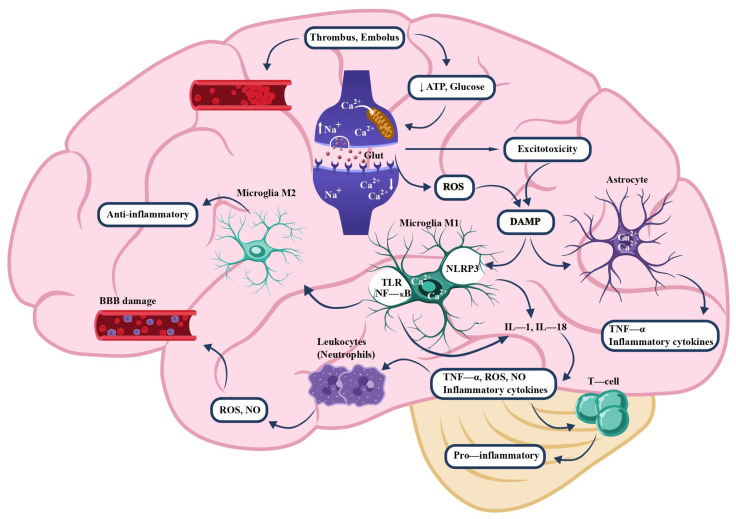
TNF-α production during stroke. Brain injury leads to a decrease in adenosine triphosphate (ATP), which disrupts the resting membrane potential and causes depolarization due to impaired Na^+^/K^+^-ATPase pump function. The accumulation of cations contributes to depolarization at nerve endings, leading to the activation of Na^+^ channels and further buildup of positive charge in neuronal terminals. The influx of Ca^2+^ ions triggers the release of neurotransmitters/glutamate into leads the synaptic cleft and/or the release of TNF-α from neuronal cells. Enhanced glutamate activity to excitotoxicity and excessive Ca^2+^ accumulation, resulting in mitochondrial damage and the production of reactive oxygen species (ROS). This activates microglia and astrocytes, which release TNF-α and other cytokines, as well as matrix metalloproteinases (MMPs) at the endothelial surface, enabling leukocyte infiltration. Abbreviations: ATP—adenosine triphosphate; BBB—blood–brain barrier; DAMP—damage-associated molecular pattern; IL-1—interleukin-1 (most commonly IL-1β); IL-18—interleukin-18; NF-κB—nuclear factor kappa-light-chain-enhancer of activated B cells; NLRP3—NOD-like receptor family pyrin domain containing 3; NO—nitric oxide; ROS—reactive oxygen species; T cell—T lymphocyte; TLR—toll-like receptor; TNF-α—tumor necrosis factor alpha.

The pathomechanism of ischemic stroke is a complex, multistage process involving abrupt energetic failure, excitotoxicity, oxidative stress, and activation of innate and adaptive immune mechanisms (Figure 3). An embolus or thrombus within a cerebral vessel leads to a sudden reduction in blood flow and, consequently, to deprivation of oxygen and glucose in a defined brain region [46,47,48]. Consequently, adenosine triphosphate (ATP) production rapidly declines, causing anoxic membrane depolarization [48,49,50], tissue acidosis, cytotoxic edema, and necrotic cell death within the infarct core [51,52].

Excessive Ca^2+^ influx increases vesicular release of neurotransmitters and gliotransmitters, primarily glutamate and TNF-α, thereby promoting neuronal injury. Overactivation of glutamatergic receptors induces excitotoxicity and Ca^2+^ overload, leading to mitochondrial dysfunction, release of pro-apoptotic factors, and initiation of apoptotic pathways [46,48,53,54]. Mitochondrial damage is accompanied by increased ROS production, further contributing to neuronal apoptosis and necrosis [55,56].

ROS and reactive nitrogen species (RNS), including those generated by excessive nitric oxide (NO) synthesis, activate microglia and astrocytes, triggering the release of inflammatory mediators such as TNF-α, IL-1β, IL-6, chemokines (neutrophil-recruiting chemokine (CXCL1), monocyte-chemoattractant chemokine (CCL2)), free radicals, and matrix metalloproteinases (MMPs) [46,57,58,59].

During the acute phase of stroke (within several hours to 3 days), this inflammatory response is rapidly amplified. Damaged neurons release danger-associated molecular patterns (DAMPs), including high mobility group box 1 (HMGB1), which further stimulate innate immune signaling [60,61].

Simultaneously, expression of nitric oxide synthases (neuronal NO synthase (nNOS), endothelial NO synthase (eNOS), and inducible NO synthase (iNOS)) increases. Excess NO reacts with superoxide (O_2_^•−^) to form peroxynitrite (ONOO^−^), a highly reactive oxidant that induces protein nitration, DNA damage, and lipid peroxidation. Peroxynitrite and other reactive species degrade tight-junction proteins such as occludin, claudin-5, and ZO-1, leading to endothelial dysfunction and increased BBB permeability [62,63].

Under the influence of cytokines and chemokines, endothelial cells upregulate adhesion molecules, facilitating leukocyte recruitment, primarily neutrophils [57,64].

Neutrophils release elastase, myeloperoxidase (MPO), and matrix metalloproteinase-9 (gelatinase B/MMP-9) as well as matrix metalloproteinase-2 (gelatinase A/MMP-2), which collectively degrade components of the basement membrane and tight junctions, thereby exacerbating blood–brain barrier damage, promoting brain edema, and increasing susceptibility to hemorrhagic transformation. In parallel, TNF-α enhances the activity of matrix metalloproteinase-3 (stromelysin-1/MMP-3) and MMP-9, further compromising barrier integrity [65,66].

The NOD-like receptor family, pyrin domain containing 3 (NLRP3) inflammasome, plays a key role in the innate immune response [67,68,69]. Toll-like receptor signaling induces NF-κB activation, promoting transcription of pro-IL-1β and pro-NLRP3 (signal 1) [5,46,70]. In the subsequent step (signal 2), triggered by ROS, ATP, and changes in membrane potential, NLRP3 undergoes oligomerization, activating caspase-1, which converts pro-IL-1β and pro-IL-18 into their active forms and initiates pyroptosis [5,67,69].

Activated microglia adopt the M1 phenotype, characterized by the release of pro-inflammatory cytokines (TNF-α, IL-1β, IL-6, interleukin-18 (IL-18)), reactive oxygen species (ROS), and matrix metalloproteinases (MMPs), which potentiate neurotoxicity. In the later phase of the response, a subset of microglia shifts toward the M2 phenotype, predominantly secreting anti-inflammatory cytokines (interleukin-10 (IL-10), interleukin-4 (IL-4), and transforming growth factor beta (TGF-β)), which facilitate reparative processes, angiogenesis, and tissue remodeling [57,70].

Importantly, microglia in the peri-infarct zone may exert neuroprotective effects by phagocytosing neutrophils and cellular debris, thereby attenuating the local inflammatory response [71,72]. This phagocytic activity is considered neuroprotective, limiting the destructive potential of neutrophils and preventing further vascular and parenchymal damage [73,74,75].

Cells of the adaptive immune response, including monocytes and T lymphocytes, infiltrate the brain at later stages, further amplifying the secondary inflammatory response and contributing to tissue injury. Concurrently, stroke induces post-stroke immunosuppression, characterized by lymphopenia and T-cell dysfunction, which increases susceptibility to infections [58,76].

TNF-α plays a particularly important role, mainly of microglial origin [71].

Its effects are highly time-dependent and determined by the specific receptor engaged. In the acute phase, high concentrations of TNF-α preferentially activate TNFR1, promoting apoptosis, necroptosis, and activation of c-Jun N-terminal kinase (JNK)/p38 mitogen-activated protein kinase (MAPK), as well as NF-κB signaling pathways, thereby exacerbating neuronal injury. In the subacute phase (days to weeks), as TNF-α levels decline, TNFR2 activation on neurons, progenitor cells, and stem cells becomes more prominent, thereby supporting neurogenesis, synaptic plasticity, and tissue regeneration [77,78].

Moderate TNFR1 activation in neurons can also exert preconditioning effects. Exposure to low TNF-α concentrations for 24–48 h enhances neuronal resistance to subsequent ischemic episodes by inducing antioxidant enzymes, heat-shock proteins, and apoptosis inhibitors [79,80].

Studies using TNF knockout mice have demonstrated that the complete absence of TNF-α augments neuronal susceptibility to ischemic injury, suggesting that basal TNF-α levels are essential for maintaining neuronal homeostasis [81,82,83,84].

Conversely, genetic or pharmacological selective ablation of the soluble form of TNF-α has been shown to exert neuroprotective effects in acute ischemic stroke, indicating that TNF-α functions are phase- and context-dependent. While excessive sTNF release exerts cytotoxic effects, constitutive low-level TNF-α signaling supports neuronal survival [85,86,87,88,89].

Elevated levels of the soluble form of TNF-α are characteristic not only of acute and chronic neuroinflammation but also of a broad spectrum of neurodegenerative disorders, including ischemic stroke, Alzheimer’s disease, Parkinson’s disease, amyotrophic lateral sclerosis, and multiple sclerosis [90,91,92]. This observation underscores the complex and dual role of TNF-α in the brain, and elucidating the interplay among ischemia, excitotoxicity, mitochondrial oxidative stress, inflammasome activation, and the dynamic contribution of the immune system is essential for the development of novel therapeutic strategies that integrate neuroprotective interventions in the acute phase with the promotion of repair processes and neuronal plasticity in the subacute and chronic phases [77,78].

## 4. TNF-α as a Biomarker in Ischemic Stroke

Tumor necrosis factor-alpha is a key pro-inflammatory cytokine that intensifies neuronal injury through mechanisms such as apoptosis, the promotion of inflammatory cell infiltration, and disruption of the blood–brain barrier. TNF-α has been found to exhibit both neurotoxic and neuroprotective effects in stroke [93].

There are conflicting results regarding TNF-α as an inflammatory mediator for predicting outcome and infarct size. Many studies confirm the occurrence of elevated TNF levels in blood and cerebrospinal fluid (CSF) in patients with acute ischemic stroke (Table 1).

Zaremba and Losy conducted one of the earliest studies examining the relationship between TNF-α levels and the severity of ischemic stroke as well as neurological outcomes [94]. They reported significantly elevated TNF-α concentrations in both cerebrospinal fluid (CSF) and serum within the first 24 h following the onset of acute ischemic stroke. Furthermore, these studies demonstrated that baseline TNF-α levels in CSF and serum correlate with early neurological symptom severity and functional disability in stroke patients [94]. Examinations of patients were performed using the Scandinavian Stroke Scale (SSS) and the Barthel Index (BI) within 24 h of stroke onset and at the 1st and 2nd weeks after onset of symptoms. The significant correlation between increased TNF-α levels and the neurological stroke severity, as well as between TNF-α levels and the degree of functional disability in stroke patients, was observed. These studies have demonstrated TNF-α’s role in mechanisms of early stroke-induced inflammation and its predictive value for stroke course.

Vila et al., in turn, showed elevated TNF-α levels in CSF and serum of stroke patients whose condition deteriorated, but the differences observed did not correlate with early worsening, which questioned Zaremba’s results [95]. TNF-α levels were significantly higher in patients who deteriorated than in those who remained stable or improved during the first 48 h. However, this study did not include healthy controls, which hampers the direct comparison with the other studies. The severity of stroke was scored on admission and after 48 h using the Canadian Stroke Scale (CSS).

Other studies have shown that TNF-α levels were elevated in patients at the sixth hour after ischemic stroke onset compared with controls, and TNF-α did not correlate with lesion size or neurologic outcome [96]. There was no significant difference in TNF-α levels between patients with severe stroke (National Institutes of Health Stroke Scale (NIHSS) ≥ 15) and those with mild or moderate stroke (NIHSS < 15).

Castellanos et al. investigated the potential association between elevated TNF-α levels in blood and a poor prognosis in lacunar infarctions. TNF-α concentrations were significantly higher in patients with lacunar infarctions (LACI) located at the basal ganglia and brainstem than in those with normal computed tomography (CT)/magnetic resonance imaging (MRI) or lacunar infarctions located in the white matter [97].

Sotgiu et al. evaluated the serum concentrations of a panel of biomarkers known to be variably associated with ischemic stroke in a consecutive series of patients with acute brain ischemia without concomitant infections, and to correlate their level with the extent of brain lesion as documented by CT and neurological outcome both at study entry and at a 3-month period [98]. The serum levels of TNF-α were measured in both stroke and other neurological disease (OND) patients, and were found to be higher in the stroke group. Furthermore, TNF-α showed a significant direct correlation with the initial and final NIH scores and infarct size.

The Tuttolomondo group investigated some differences in plasma cytokine levels (including TNF-α) in the acute phase of stroke depending on Trial of Org 10172 in Acute Stroke Treatment (TOAST) ischemic stroke subtype [99,100]. The lower magnitude of cytokine activation in the acute phase may be related to the limited extent of lacunar strokes, with a possible association between reduced TNF-α and IL-1β plasma levels in the acute phase and the milder acute neurological deficit characteristic of the lacunar subtype. Patients with lacunar stroke, compared to other subtypes, had a lower degree of acute neurological deficit on admission, evaluated by SSS, and a lower degree of immuno-inflammatory activation of the acute phase. On the other hand, cardioembolic strokes, in comparison with other subtypes, showed a higher grade of acute neurological deficit at admission evaluated by SSS, and a higher grade of immunoinflammatory activation of the acute phase [100]. This suggests that cardioembolic strokes have a poorer prognosis and cause larger and more disabling strokes than other ischemic stroke subtypes [101]. Moreover, diabetic patients with lacunar strokes exhibited a minor grade of immunoinflammatory activation of the acute phase at 24–72 h and 7–10 days after stroke onset. This minor grade of immunoinflammatory activation may be related to the minor extension of infarct size, due to the typical microvascular disease in diabetic subjects. It was also found that patients with lacunar stroke and no detectable lesion on brain CT or MRI do not show any significant difference in acute-phase cytokine plasma levels compared to patients with a detectable lacunar lesion (on CT or MRI) [100,102]. An analogous relationship between TNF-α levels and stroke subtype was reported by Góngora-Rivera et al. [103]. However, these studies lack data regarding the control group. The lowest levels of this cytokine were observed in cases of lacunar infarction, but did not correlate with a worse functional outcome upon hospital discharge or at 3 months following discharge. Furthermore, a positive correlation was found between intima-media thickness greater than 1 mm and TNF-α. TNF-α was not associated with the extension of the stroke by an ASPECTS score with CT or MRI, or with the arterial territory involved.

Intiso et al., however, reported no significant differences in TNF-α levels in patients with different types of ischemic stroke (although the levels were higher in small-vessel infarction (SI) compared to large-artery infarction (LaI) and cardioembolic infarction (CEI)), but also observed a notable increase in TNF-α levels compared to the control group [104]. Mean baseline TNF-α levels in the stroke group did not differ from those in the control group. A statistically significant increase in TNF-α levels was observed over the course of the study, with concentrations rising during the first week after admission and subsequently declining, while remaining elevated relative to baseline values at the later time point. Male patients tended to exhibit higher TNF-α levels than female patients at both admission and follow-up; however, these sex differences did not reach statistical significance. In addition, peak TNF-α concentrations were comparable between patients with and without infectious complications. Nevertheless, TNF-α levels in both subgroups were consistently higher than those observed in the control group.

In turn, Nakashidze et al. reported that TNF-α levels were significantly elevated in male patients with ischemic stroke and suggested that sex hormones may modulate TNF-α production, with androgens potentially enhancing TNF-α expression and estrogens exerting a suppressive effect [105]. This may explain why men show higher TNF-α responses and more severe inflammation after stroke [106].

Vlădoiu and Moga presented studies on patients diagnosed with acute ischemic stroke, evaluating TNF-α levels on days 1 and 7 post symptom onset; however, there was no comparison to the control group [107]. Elevated TNF-α levels measured at both time points were significantly associated with greater stroke severity and poorer functional outcomes, as reflected by higher NIHSS and modified Rankin Scale (mRS) scores. Overall, TNF-α concentrations declined significantly between the early and later assessments, indicating a dynamic post-stroke inflammatory response. Importantly, despite this general decrease over time, relatively higher TNF-α levels at the later time point remained correlated with greater neurological deficit, suggesting that persistent TNF-α elevation may be linked to more severe disease and unfavorable recovery trajectories.

Kashyap et al. also demonstrated a significant increase in patients with acute ischemic stroke at 24 h and 48 h, followed by a gradual decrease at 72 h, 144 h, and at discharge [108].

Studies conducted by our research group demonstrated elevated serum TNF-α levels in the acute phase of ischemic stroke, with a subsequent decline by the seventh day after symptom onset [109]. TNF-α concentrations were consistently higher in stroke patients compared with control subjects. Importantly, analysis across different ischemic stroke subtypes revealed that patients with LACI exhibited the lowest TNF-α levels when assessed within the early therapeutic time window, a finding that is consistent with previous reports [101,102,110]. This observation likely reflects fundamental differences in pathophysiology, extent of tissue injury, prognosis, and clinical presentation between lacunar strokes and other ischemic infarct types [111]. Moreover, significant differences in TNF-α levels were observed between LACI and posterior circulation infarction (POCI) at stroke onset and on the first day after symptom onset, further underscoring the influence of stroke subtype on the inflammatory response.

On the other hand, several studies have reported contrasting findings, in which TNF-α levels during the acute phase of ischemic stroke were compared between stroke patients and control subjects and were found to be comparable or even reduced. For instance, Luvizutto’s group reported low circulating TNF-α levels but elevated concentrations of soluble TNF receptors (TNFR1 and TNFR2) in patients with acute ischemic stroke [112]. Simultaneously, there were no differences in sTNFR1 and sTNFR2 levels between the groups. They found that there is a strong production of cytokines, including IL-10 and sTNFR2, during the first 72 h after stroke, and this increase may serve as a modulatory mechanism of local inflammation during the ischemic process. Both cytokines (IL-10 and sTNFR2) were positively correlated with infarct size thus the higher the circulating levels of IL-10 and sTNFR2, the larger the infarct size. Consistent with these observations, studies by Emsley and colleagues demonstrated that circulating TNF-α levels did not differ significantly from those in control subjects [113]. In contrast, peak levels of sTNFR1 during the first week after ischemic stroke showed strong correlations with infarct volume and long-term functional outcomes, including disability and functional independence assessed at both intermediate and long-term follow-up. These findings suggest that sTNFR1 may better reflect disease severity and prognosis than TNF-α itself. Plasma TNF-α and sTNFR2 concentrations exhibited a temporal profile similar to that of sTNFR1, although the observed changes were less pronounced. Peak levels of TNF-α, sTNFR1, and sTNFR2 were strongly interrelated, indicating coordinated activation of the TNF signaling axis following ischemic stroke. Notably, the stronger association of sTNFR1 with stroke severity and clinical outcome may be attributable to its longer circulating half-life and greater temporal stability compared with TNF-α, supporting its potential superiority as a systemic biomarker of TNF pathway activation. Fassbender et al. also observed no changes in plasma TNF-α levels immediately after or following ischemic stroke [114]. Furthermore, no differences in TNF-α concentrations were found depending on the patient’s gender.

Lambertsen et al. reported an early increase in circulating TNFR1 and TNFR2 levels during the acute phase of ischemic stroke, within hours of symptom onset, compared with healthy controls, whereas plasma TNF-α concentrations remained unchanged [115]. At 72 h after ischemic stroke, TNFR1 levels returned to values comparable to controls, while TNFR2 expression remained markedly elevated, indicating a sustained and receptor-specific activation of the TNF signaling axis during the post-ischemic period.

**Table 1:** The value of TNF-α in human ischemic stroke.

Biological Material/Collection Time After Stroke	Average Concentration	Control	Results	Conclusions	Ref.
CSF/within 24 h Serum/within 24 h	9.1 ± 5.8 pg/mL 14.0 ± 10.2 pg/mL *p* < 0.05 (*n* = 23)	6.6 ± 0.5 pg/mL 9.1 ± 1.6 pg/mL *p* < 0.05 (*n* = 15)	The levels of TNF-α in CSF were inversely correlated with the SSS-1 scores (r = −0.77; *p* < 0.0001, the SSS-2 scores (r = −0.80; *p* < 0.00001), and the SSS-3 scores (r = −0.82; *p* < 0.00001). The levels of TNF-α in serum were inversely correlated with the SSS-1 scores (r = −0.76; *p* < 0.0001), the SSS-2 scores (r = −0.77; *p* < 0.0001), and the SSS-3 scores (r = −0.77; *p* < 0.0001). The levels of TNF-α in CSF were inversely correlated with the BI-1 scores (r = −0.84; *p* < 0.00001), the BI-2 scores (r = −0.85; *p* < 0.000001), and the BI-3 scores (r = −0.87; *p* < 0.000001). The levels of TNF-α in serum were inversely correlated with the BI-1 scores (r = −0.81; *p* < 0.00001), the BI-2 scores (r = −0.82; *p* < 0.00001), and the BI-3 scores (r = −0.83; *p* < 0.00001).	An increase in TNF-α levels in CSF and serum of stroke patients within the first 24 h after the onset of stroke was observed. These results demonstrated that initial TNF-α concentrations in CSF and serum reflect the early severity of neurological symptoms and functional disability in stroke patients.	[94]
CSF/on admission	22.8 (15.4) pg/mL *p* = 0.001 (*n* = 83, neurological worsening)	no data	Eighty-three patients (35.9%) worsened within the first 48 h after stroke onset: 41 (17.7%) worsened by 1 point, 24 (10.4%) by 2 points, and 18 (7.8%) by ≥3 points. In 69 patients (29.9%), no changes were detected in the CSS score. Seventy-nine patients (34.2%) improved their CSS score within the first 48 h: 54 (23.4%) improved by 1 point, 21 (9.1%) by 2 points, and 4 by ≥3 points.	Even though TNF-a was higher in patients with early clinical worsening, the relationship was confounded by other factors, because it did not remain statistically significant on multivariate testing.	[95]
	11.1 (11.1) pg/mL *p =* 0.001 (*n* = 148, no neurological worsening)				
Serum/on admission	21.1 (8.1) pg/mL *p* < 0.0001 (*n* = 83, neurological worsening)				
	15.1 (6.1) pg/mL *p* < 0.0001 (*n* = 148, no neurological worsening)				
CSF/6 h	44 ± 5.4 pg/mL (Group 1, *n* = 44) 39.4 ± 9.4 pg/mL (Group 2, *n* = 51) *p* < 0.05	14 ± 2.3 pg/mL *p* < 0.05 (*n* = 25)	Group 1: NIHSS score at admission 20.2 (4.1); NIHSS score on 7th day 18.5 (3.2), *p* < 0.001. Group 2: NIHSS score at admission 8.6 (4.9); NIHSS score on 7th day 7.3 (3.5), *p* < 0.001.	The study did not show any significant group differences in the TNF-α CSF levels at 6 h of ischemic stroke. However, the absolute number of these cytokines was elevated in the severe stroke group, suggesting that they are of the first pro-inflammatory response and may trigger the subsequent proinflammatory cascade.	[96]
Serum/within 24 h	8.2 (6.4, 15.3) pg/mL *p* = 0.001 (*n* = 113)	7.0 (5.7, 8.4) pg/mL *p* = 0.001 (*n* = 43)	TNF-α [11.5 pg/mL (7.8 and 16.2 pg/mL) versus 7.6 pg/mL (6.2 and 13.3 pg/mL), *p* < 0.01] concentrations were significantly higher in patients with lacunar infarctions located at the basal ganglia and brainstem than in those with normal CT/MRI or lacunar infarctions located at the white matter. Plasma TNF-α > 14 pg/mL (OR, 3.0; 95% CI, 1.0 to 8.5; *p =* 0.042) and baseline CSS score (OR, 0.48; 95% CI, 0.29 to 0.79; *p* = 0.004) were independently associated with poor outcome at 3 months.	Plasma TNF-α concentrations were significantly higher in patients with lacunar infarctions than in the control group. Logistic regression analysis revealed that plasma TNF-α levels greater than 14 pg/mL were significantly associated with neurological deterioration, independent of a history of arterial hypertension, leukocyte count, and infarct location.	[97]
Serum/within 20 h	75 (37.4; 137.9) pg/mL *p* = 0.001 (*n* = 66)	37.9 (36.1; 47.2) pg/mL *p* = 0.001 (OND, *n* = 32)	TNF-α concentration at study entry correlated better with NIHSS than GCS and GOS scales; in particular TNF-α directly correlated with NIHSS scores (r Ľ 0.82, *p* < 0.001), especially in the subgroup with worse outcome (median 90, percentiles 36 and 518 pg/mL) when compared with the subgroup with better outcome (median 37.9, percentiles 35.6 and 150 pg/mL; *p* Ľ 0.002). As for the infarct size, a direct correlation was also found (r ¼ 0.92 in patients with a worse outcome and 0.77 in those with a better outcome, *p* ¼ 0.04 and 0.0002, respectively).	Elevated TNF-α levels showed a significant correlation with the clinical severity and the extent of the brain infarct.	[98]
Serum/24–72 h	37.5 (10.25–41) pg/mL *p* < 0.001 (*n* = 60) 27.5 (13.4–40.5) pg/mL *p* < 0.0001 (LAAS, *n* = 50) 19.4 (9–23) pg/mL *p* < 0.0001 (Lacunar, *n* = 46) 38.5 (22.2–46) pg/mL *p* < 0.0001 (CEI, *n* = 20) 29 (10.4–39.0) pg/mL *p* < 0.0001 (ODE, *n* = 4)	3.7 (1.1–4.3) pg/mL *p* < 0.001 (*n* = 123)	Patients with lacunar stroke in comparison with subjects with non-lacunar stroke exhibited, 24–72 h after stroke onset, lower plasma levels of TNF-α [21.8 pg/mL (18–30) vs. 33.5 pg/mL (15.25–40), *p* = 0.001]. At 7–10 days after stroke onset, TNF-α [21.8 pg/mL (18–30) vs. 33.5 pg/mL (25.25–38), *p =* 0.001] plasma levels remained lower in patients with lacunar stroke. In diabetic patients with lacunar stroke, 7–10 days after stroke onset, plasma levels of TNF-α [24.5 pg/mL (10.5–31.2) vs. 35.2 pg/mL (22–41.5) vs. 26.7 pg/mL (21.2–33.0) vs. 31.4 pg/mL (21.7–34.77), *p* = 0.001] remained significantly lower in comparison with non-diabetic patients with lacunar stroke and with diabetics and non-diabetics with non-lacunar stroke. It was found a significant association between SSS score at admission and diagnostic subtype: lacunar (b = 3.206; *p* = 0.0338) or cardioembolic (b = −7.819; *p* = 0.0006) and some inflammatory variable TNF-α (b = −0.013; *p* < 0.0001) or IL-6 (b = −0.074; *p* < 0.0001).	Higher plasma TNF-α levels were observed in stroke patients in comparison with control subjects without acute ischemic stroke.	[100,101,102]
Serum/0–16 days (56% within 48 h)	6 (0.79–17.38) pg/mL (*n* = 75)	no data	For small-vessel disease or lacunar infarct, lower NIHSS scores were observed, indicating less neurological damage (*p* = 0.044). With the modified Rankin scale, the etiology with the poorest prognosis at discharge was atherothrombosis (*p* = 0.041), and small vessel disease again had the best functional prognosis, i.e., lower mRS values (*p* = 0.003). At 3 months, the etiology with the poorest prognosis was cardioembolic (*p* = 0.001), and the etiology with the best prognosis was small vessel disease (*p* < 0.0001).	Lacunar stroke was characterized by significantly lower levels of TNF-α, but this was not associated with better functional prognosis at hospital discharge and at follow-up at 3 months. An association between higher levels of TNF alpha and carotid intima-media thickness of more than 1 mm was found. The Authors claimed that more prospective studies with larger patient numbers are needed to validate these results.	[103]
	6.13 (0.9–17.38) pg/mL (Atherothrombotic, *n* = 29)				
	4.79 (0.91–16.12) pg/mL (Lacunar, *n* = 20) *p* = 0.048				
	7.37 (1.75–13.99) pg/mL (CEI, *n* = 20)				
	6.64 (1.96–15.56) pg/mL (Indeterminate origin, *n* = 15)				
Serum/on 7 day	55.9 ± 40.3 pg/mL *p* = 0.069 (*n* = 41)	29.0 ± 13.9 pg/mL *p* = 0.069 (*n* = 40)	Mean baseline TNF-α levels in the stroke group: 30.1 ± 12.5 pg/mL, *p* = 0.746. TNF-α levels on day 10: 48.0 ± 24.1 pg/mL. In male compared to female subjects, higher values of TNF-α were observed at admission: 34.5 ± 16.8 vs. 24.7 ± 9.59 pg/mL, *p* = 0.079; and on day 10: 56.4 ± 30.7 vs. 40.9 ± 13.9 pg/mL, *p* = 0.056. TNF-α levels in patients with infectious complications and those without infectious complications: 47.1 ± 16.8 vs. 63.4 ± 52.0 pg/mL).	Serum TNF-α levels showed an early and prolonged increase after stroke onset, unrelated to lesion size, neurological impairment, age, sex, vascular risk factors, or infectious complications. The serum increase in TNF-α may be part of the acute-phase response observed in stroke patients.	[104]
	57.5 ± 21.2 pg/mL (Lacunar, *n* = 10) *p* = 0.069				
	66.9 ± 53.8 pg/mL (Subcortical, *n* = 14) *p* = 0.069				
	44.4 ± 35.3 pg/mL (Cortical, *n* = 17) *p* = 0.069				
Serum/on 1 day, on 7 days	7.39 (4.9–10.96) pg/mL (*n* = 56) *p* = 0.01 on 1 day	no data	The mean NIHSS score at admission was 14, indicating a moderate to severe stroke population. On day 7, the average NIHSS score dropped to 10.2. A positive correlation was observed between TNF-α level and mRS scores at discharge (on 1 day, r = 0.719, *p* < 0.001, on 7 days, r = 0.823, *p* < 0.001). The ROC analysis: TNF-α on 1 day showed an AUC of 0.907 (95% CI: 0.819–0.995).	Significant correlations were observed between IL-6 and TNF-α, stroke severity as measured by the NIHSS, and disability outcomes as assessed by the modified Rankin Scale, highlighting their potential as reliable predictors of both acute stroke and long-term recovery.	[107]
	5.12 (3.9–9.34) pg/mL *p* < 0.05 on 7 day				
Serum/within 4.5 h, within 24 h, within 7 days	42.4 (32.2–50.9) pg/mL (*n* = 125) *p* < 0.001 within 4.5 h	29.8 (27.8–31.8) pg/mL (*n* = 28) *p* < 0.001 within 4.5 h	The relationship between the TNF-α levels measured during onset and the NIHSS on admission (r = 0.4, *p* = 0.02) as well as mRS values assessed on admission (r = 0.33, *p* = 0.01) and in the 3rd month since the stroke (r = 0.47, *p* < 0.01) was observed. Good sensitivity and specificity were found in the TNF-α levels’ assessment < 4.5 h and on the 1st day since the stroke during evaluation of the patients’ neurologic disability; after 3 months (<4.5 h: cut-off point = 39.94 pg/mL, sensitivity = 62.8%, specificity = 100%, AUC = 0.840; after a day: cut-off point = 60.14 pg/mL, sensitivity = 96.7%, specificity = 50.0%, AUC = 0.733) and 1 year (<4.5 h: cut-off point = 42.54 pg/mL, sensitivity = 62.8%, specificity = 100%, AUC = 0.832; after a day: cut-off point = 60.14 pg/mL, sensitivity = 60.14%, specificity = 100%, AUC = 0.966) since the stroke.	A relationship between TNF-α level and the severity of the neurological deficit was observed. An association between TNF-α and functional outcomes was demonstrated in a group of patients without prior infection and other chronic inflammatory diseases, indicating that TNF-α is an inflammatory marker of acute ischemic brain injury.	[109]
	46.2 (34.0–52.3) pg/mL (*n* = 125) *p* < 0.001 within 24 h				
	33.9 (31.5–50.0) pg/mL (*n* = 125) *p* < 0.001 within 7 days				
Plasma/within 72 h	0.0 (0.0–0.0) pg/mL (*n* = 15) *p* = 0.0036	0.23 (0.0–2.4) pg/mL (*n* = 20) *p* = 0.0036	sTNFR2 was positively correlated with infarct size (r = 0.689, *p* = 0.018), therefore the higher the levels of circulating sTNFR2, the worse the infarct size.	The results indicate that the plasma levels of some cytokines may be associated with changes in the acute phase of stroke; therefore, sTNFR2 level can be considered a potential biomarker of infarct size.	[112]
	sTNFR1 499.3 (293.2–580.9) pg/mL (*n* = 15) *p =* 0.581	sTNFR1 517.1 (293.5–702.2) pg/mL (*n* = 20) *p* = 0.581			
	sTNFR2 525.8 (450.3–626.8) pg/mL (*n* = 15) *p* = 0.773	sTNFR2 526.6 (415.5–676.1) pg/mL (*n* = 20) *p* = 0.773			
Plasma/within 8 h	2.0 (0.7–2.6) pg/mL (*n* = 34) *p* = 0.57	1.9 (1.6–2.6) pg/mL (*n* = 28) *p* = 0.57	TNF IR was initially found in neurons located in I/PI and NAT, but increased in glia in older infarcts. TNF IR increased in macrophages in all specimens. TNFR1 IR was found in neurons and glia and macrophages, while TNFR2 was expressed only by glia in I/PI and NAT, and by macrophages in I/PI. These results suggest that TNF and IL-1 are expressed by subsets of cells and that TNFR2 is expressed in areas with increased astrocytic reactivity.	The findings of increased brain cytokines and plasma TNFR1 and TNFR1 support the hypothesis that targeting poststroke inflammation could be a promising add-on therapy in ischemic stroke patients.	[115]
	sTNFR1 376.4 (146.8–320.2) pg/mL (*n* = 34) *p* = 0.006	sTNFR1 131.9 (118.5–158.7) pg/mL (*n* = 28) *p* = 0.006			
	sTNFR2 413.8 (251.3–709.0) pg/mL (*n* = 34) *p* = 0.04	sTNFR2 276.7 (236.7–357.8) pg/mL (*n* = 28) *p* = 0.04			
Plasma/72 h	2.0 (1.8–3.1) pg/mL (*n* = 9) *p* = 0.57	1.9 (1.6–2.6) pg/mL (*n* = 28) *p* = 0.57			
	sTNFR1 303.3 (141.0–193.6) pg/mL (*n* = 9) *p* = 0.006	sTNFR1 131.9 (118.5–158.7) pg/mL (*n* = 28) *p* = 0.006			
	sTNFR2 518.7 (266.7–660.5) pg/mL (*n* = 9) *p* = 0.04	sTNFR2 276.7 (236.7–357.8) pg/mL (*n* = 28) *p* = 0.04			

BI—BarthelI; CEI—cardioembolic infarct; CSF—cerebrospinal fluid; CSS—Canadian Stroke Scale; CT—computed tomography; GCS—Glasgow Coma Scale; GOS—Glasgow Outcome Scale; IL-1—interleukin-1; IL-6—interleukin-6; I/PI—ipsilateral/peripheral infarction; MRI—magnetic resonance imaging; mRS—modified Rankin Scale; NAT—neuroaxonal damage; NIHSS—National Institutes of Health Stroke Scale; LAAS—large artery atherosclerosis; OND—other neurological disorders; ODE—other determined etiology; sTNFR1—soluble tumor necrosis factor receptor 1; sTNFR2—soluble tumor necrosis factor receptor 2; SSS—Scandinavian stroke scale; TNF-α—tumor necrosis factor alpha; TNFR1—tumor necrosis factor receptor 1.

Discrepancies in study findings regarding the role of TNF-α in ischemic stroke arise from the multidimensional, highly context-dependent nature of its biological activity, as well as from substantial methodological differences across studies. One key determinant of inconsistent observations is the timing of biological sample collection. TNF-α exhibits a highly dynamic expression profile following an ischemic event, with a rapid increase in circulating levels during the first hours, followed by a rapid decline or secondary fluctuations during the subacute and chronic phases. However, relatively few clinical studies in humans assess peripheral cytokine responses within the first 4 h after stroke onset, a critical time window for infarct progression, during which early inflammatory events may simultaneously contribute to secondary tissue injury and influence reparative processes. Consequently, the time point at which TNF-α is measured is one of the main factors that differentiate experimental and clinical study outcomes [93]. In addition, stroke severity affects both the magnitude and temporal dynamics of TNF-α elevation, underscoring the importance of early cytokine profiling for prognostic assessment and the identification of potential therapeutic interventions [93]. As a result, studies relying on single time point measurements, particularly those collected far from symptom onset, may yield conflicting conclusions about the association between TNF-α levels, stroke severity, and clinical outcomes.

Another major source of inconsistency is the heterogeneity of ischemic stroke itself. Different pathophysiological subtypes, such as lacunar, cardioembolic, or large-artery atherosclerotic stroke, vary in lesion size, degree of blood–brain barrier disruption, and inflammatory response profiles. A general trend toward lower serum TNF-α concentrations has been reported in patients with lacunar stroke related to small-vessel cerebral microangiopathy compared with other ischemic stroke etiologies [101,102,109,110]. These differences substantially limit the direct comparability of results obtained from heterogeneous patient cohorts. Moreover, regional population differences, diagnostic criteria, neuroimaging methodologies, access to healthcare, and the level of technological advancement used for biomarker measurements further influence data interpretation [116].

In addition, only a limited number of clinical stroke studies have sufficient statistical power to perform stratified analyses by stroke location or severity, which significantly contributes to the observed variability in cytokine levels [93].

Methodological differences in TNF-α detection also contribute significantly to discrepant findings. The use of assays with varying sensitivity and specificity, failure to distinguish between soluble TNF-α and transmembrane TNF, and the omission of concurrent measurements of soluble TNF receptors (sTNFR1 and sTNFR2) introduce substantial interpretative challenges. An increasing body of evidence suggests that circulating levels of sTNFR1 and sTNFR2 may better reflect long-term TNF-α axis activity than sTNF-α alone, whose short half-life and predominantly local mode of action limit its utility as a stable systemic biomarker. At the same time, elevated sTNFR concentrations may reflect both inflammatory activation and compensatory regulatory mechanisms, complicating the biological interpretation of changes in these levels [82,113,117].

An additional confounding factor is the presence of comorbid conditions such as diabetes mellitus, obesity, arterial hypertension, or chronic inflammatory diseases, which independently modulate TNF-α signaling and may substantially distort observed associations in acute ischemic stroke [118,119]. In many clinical studies, these variables are not fully controlled for or analyzed as confounders. Furthermore, control groups in studies of inflammatory mediators in stroke are often heterogeneous, ranging from healthy volunteers to individuals with comorbidities, further limiting comparability. Consequently, using a single inflammatory mediator, such as TNF-α, to predict infarct size or clinical outcome is insufficient given the substantial heterogeneity of peripheral cytokine responses [93].

Despite these limitations, an increasingly clear consensus has emerged in the literature that the role of TNF-α in ischemic stroke is strictly dependent on timing, receptor subtype, and tissue context. Early activation of the sTNF–TNFR1 axis is widely recognized as a mechanism that exacerbates secondary ischemic injury, whereas TNFR2-dependent signaling plays a crucial role in later reparative and adaptive phases. TNFR1 activation triggers multiple signaling pathways, including NF-κB and MAPK cascades, which regulate cell survival and death, immune responses, and inflammation. Importantly, recent studies highlight a context-dependent, dual role of TNFR1, which, depending on the timing of activation, microenvironmental conditions, and interaction with other signaling molecules, may promote either cell survival or apoptosis [30,120]. In this context, key unresolved questions concern the identification of precise therapeutic time windows in which TNF-α modulation would be most beneficial, as well as whether a combined assessment of sTNF-α, together with soluble receptors sTNFR1 and sTNFR2, more accurately reflects the dynamic balance between neuroinflammatory processes and reparative mechanisms after ischemic stroke than single-marker measurements.

From a clinical perspective, the principal barriers to using TNF-α as a diagnostic or prognostic tool include its strong dependence on time from stroke onset, stroke subtype, and comorbid disease burden, as well as the lack of standardized measurement methods and the limited availability of rapid, multiparametric assays applicable within the acute therapeutic window [93,109,116]. Consequently, isolated TNF-α measurements have limited clinical utility. Future clinical strategies will likely require integrating the TNF-α axis into multidimensional biomarker panels alongside neuroimaging and clinical parameters, rather than relying on a single indicator [82,117]. Such an approach may enable more precise patient stratification, improved prognostic accuracy, and personalized therapeutic interventions in ischemic stroke, potentially translating into improved rehabilitation trajectories and reduced long-term disability [107].

For these reasons, further experimental and clinical studies are required to precisely define the conditions under which immunomodulatory interventions can improve post-stroke outcomes without increasing the risk of secondary injury.

## 5. TNF-α as a Therapeutic Target

For many years, TNF-α has been recognized as one of the principal mediators of inflammation and, simultaneously, one of the most complex regulators of immune and cellular function. In the central nervous system (CNS), its actions are particularly ambivalent: the soluble form primarily activates TNFR1, thereby inducing pro-inflammatory, necrotic, and apoptotic signaling, whereas the membrane-bound form preferentially activates TNFR2, which promotes neuronal survival, remyelination, and reparative processes [15,82,92,121]. This duality implies that both excessive TNF-α activity and its non-selective neutralization may lead to adverse effects, making precisely targeted modulation of this pathway one of the most promising strategies in contemporary immunotherapy and neuroprotection.

In the first generation of TNF-targeted therapies, non-selective agents were introduced that block both soluble TNF (sTNF) and transmembrane TNF (tmTNF), thereby suppressing signaling through both TNF receptors. Etanercept, a fusion protein composed of the p75 TNFR2 extracellular domain fused to the Fc region of IgG1, neutralizes TNF-α and lymphotoxin-α and has remained a cornerstone therapy for rheumatoid arthritis, psoriasis, and ankylosing spondylitis for many years [122]. Infliximab, a chimeric IgG1 monoclonal antibody, is used in Crohn’s disease, ulcerative colitis, and psoriasis, and its anti-inflammatory efficacy has been consistently demonstrated [123,124]. Adalimumab, a fully human anti-TNF IgG1 antibody, is equally widely used and has additional applications in non-infectious uveitis [125]. Subsequent agents, such as certolizumab pegol, an Fc-free Fab fragment with distinct pharmacokinetics, and golimumab, also effectively neutralize TNF-α [126,127]. Despite their substantial clinical success, the mechanism of action of these agents is inherently non-selective, as they inhibit not only detrimental TNFR1-mediated signaling but also potentially beneficial TNFR2-dependent activity, a limitation that may be particularly relevant in the context of neuroinflammatory disorders [15,82,92,121].

For this reason, a second generation of TNF-modulating therapies has been developed, aiming not at complete TNF-α blockade but at the selective inhibition of its pathogenic pathways. One of the most promising examples is XPro1595, a dominant-negative TNF variant that selectively neutralizes soluble TNF while preserving tmTNF–TNFR2 signaling [78]. Another key candidate is Atrosimab, a monovalent TNFR1-selective antibody that selectively blocks TNFR1, thereby suppressing deleterious signaling cascades while preserving TNFR2-mediated neuroprotective functions [128]. Both molecules were designed to modulate TNF-α in a manner consistent with its physiological duality.

In a mouse model of permanent focal cerebral ischemia, systemic administration of XPro1595 improved functional neurological outcomes and modulated peripheral immune responses, but did not significantly affect infarct volume. In contrast, in a separate study employing topical administration immediately after ischemia, XPro1595 reduced infarct volume and attenuated post-stroke inflammatory responses [78,129,130,131]. Notably, the timing of anti-inflammatory therapy is critical in stroke models: treatment administered later in the therapeutic window showed significantly reduced efficacy compared with early intervention [78,85].

The mechanism underlying this dependence is well understood: in the first hours after stroke, TNF-α primarily signals through TNFR1, thereby enhancing apoptosis, excitotoxicity, and blood–brain barrier permeability [15,82,86,92,121,129,131]. However, in later phases, TNF-α assumes compensatory functions, supporting phagocytosis, angiogenesis, synaptic network remodeling, and remyelination via TNFR2 [125,132]. As a result, late TNF-α blockade also suppresses reparative processes, explaining the discrepancies observed across studies.

In demyelination models such as experimental autoimmune encephalomyelitis (EAE), inhibition of sTNF or TNFR1 improved remyelination, reduced astroglial activation, and protected axons—but only when therapy began in the early phase of the disease [132,133,134,135]. Similarly, Atrosimab displayed strong neuroprotective effects in an N-methyl-D-aspartate (NMDA) receptor–mediated injury model, but only when administered during the acute excitotoxic phase [128,129,131,136]. In a combined model of rheumatoid arthritis and induced cerebral ischemia, mice treated with TNF inhibitors prior to stroke induction showed significantly reduced infarct volumes and a more favorable microglial profile [136].

In contrast to the more consistent preclinical findings, clinical data on TNF-α in stroke remain inconclusive. Studies assessing TNF-α levels in patients during the acute phase of stroke have reported heterogeneous results: while some demonstrate an association between elevated cytokine concentrations and worse clinical outcomes, others fail to identify statistically significant differences [9]. An additional translational limitation is the absence of clinical trials evaluating selective TNFR1 and soluble TNF (sTNF) inhibitors, such as XPro1595 or Atrosimab, in central nervous system disorders, primarily due to challenges related to antibody penetration across the blood–brain barrier. It is well established that most IgG antibodies access the brain parenchyma only to a very limited extent [137,138,139,140,141,142].

Anti-TNF therapies are further constrained by risks associated with systemic immunosuppression. The best-characterized complication is increased susceptibility to infections, including reactivation of latent tuberculosis [143]. Although rare, cases of demyelination associated with anti-TNF treatment have also been documented [144].

Nevertheless, advances in CNS drug-delivery technologies are opening new possibilities for overcoming these barriers. Rapidly evolving approaches include nanoparticle-based delivery systems equipped with transport ligands, liposomal formulations, receptor-mediated transcytosis strategies, and methods for transient and controlled BBB disruption using focused ultrasound [137,138,139,140,141,142].

Translational challenges also arise from the intrinsic biological duality of TNF-α. At low concentrations or during short-term exposure, TNF-α may exert neuroprotective effects, such as the induction of molecular chaperones and the promotion of TNFR2-dependent regenerative processes, whereas excessive or sustained TNFR1 activation leads to neurotoxicity [82,88,121]. These complexities are further compounded by species-specific differences in pharmacokinetics, immune responses, and BBB structure, which complicate the direct translation of animal findings to humans [138,141].

Considering all available evidence, TNF-α remains one of the most promising yet simultaneously most challenging therapeutic targets in neurology and immunology. Strategies that selectively inhibit the pathogenic sTNF–TNFR1 axis while preserving TNFR2-mediated signaling appear particularly promising [88,128]. Coupling such molecules with advanced CNS drug-delivery platforms may ultimately enable the development of effective and precise neuroprotective therapies based on targeted TNF-α modulation.

## 6. Clinical and Translational Considerations

Despite compelling mechanistic evidence supporting selective modulation of the sTNF–TNFR1 axis (e.g., XPro1595) and receptor-specific blockade of TNFR1 (e.g., Atrosimab), translating these strategies into clinical practice faces substantial limitations. Importantly, these barriers arise directly from the biology of TNF-α described in the mechanistic sections of this work, particularly from its pronounced time- and receptor-dependent duality of action.

The first and most fundamental challenge is identifying an optimal therapeutic window. Preclinical data consistently demonstrate that during the early phase of acute central nervous system injury, pathogenic signaling mediated by the sTNF/TNFR1 axis predominates, promoting excitotoxicity, neuronal apoptosis, blood–brain barrier dysfunction, and pro-inflammatory microglial activation. As the disease process evolves, the role of TNF-α progressively shifts toward compensatory and reparative mechanisms that are more strongly dependent on TNFR2 activation, including phagocytosis, synaptic network remodeling, angiogenesis, and remyelination. Consequently, selective inhibition of sTNF or TNFR1 is expected to confer therapeutic benefit only within a restricted temporal window in which TNFR1-mediated pathogenic signaling outweighs later regenerative processes. From a translational perspective, this necessitates the design of clinical trials with explicitly defined intervention windows aligned with the biological phase of disease, rather than relying solely on arbitrary time points from symptom onset [78,82,85,132].

A second major limitation relates to the penetration of biologic agents across BBB, particularly in the case of monoclonal antibodies. Under physiological conditions, IgG-class molecules exhibit minimal access to the central nervous system, and even in acute pathological states, BBB disruption is transient and spatially heterogeneous. As a result, systemic administration may fail to achieve sufficient or uniform receptor engagement within brain tissue. Potential strategies to overcome this limitation include the use of BBB transport-enhancing systems, such as receptor-mediated transcytosis, alternative routes of administration, or approaches involving transient and controlled BBB opening. However, each of these strategies introduces additional challenges related to safety, dosing, and reproducibility. Therefore, in early-phase clinical studies, it is essential to confirm not only the presence of the therapeutic agent within the central nervous system but also its effective engagement of the intended molecular target, in order to reduce the risk of misleading efficacy readouts, particularly when CNS exposure or target engagement is uncertain [138,139,140].

A third challenge is the substantial heterogeneity in inflammatory responses among patients, which necessitates integrating biomarkers into clinical trial design. Selective anti-TNF therapies should not be evaluated in unselected patient populations, as in some individuals the sTNF–TNFR1 axis may not be the dominant pathogenic mechanism. The use of screening biomarkers, such as soluble TNF receptors, to identify patients with active TNF-dependent signaling, together with pharmacodynamic biomarkers confirming biological drug activity, may substantially increase trial sensitivity for detecting therapeutic effects. This approach enables a direct linkage between molecular mechanism, patient selection, target engagement, and interpretation of clinical efficacy [113,115,116].

We hypothesize that future precision anti-inflammatory therapies for ischemic stroke will require stratification not on absolute TNF-α levels but on dynamic biomarkers reflecting TNF pathway balance, such as the sTNFR1:sTNFR2 ratio, which may better capture the shift from TNFR1-driven injury to TNFR2-mediated repair.

## 7. Conclusions

Given the pivotal role of TNF-α in the pathophysiology of ischemic stroke, its use as both a diagnostic and prognostic biomarker, as well as a target for selective anti-inflammatory therapy, appears highly justified. Importantly, TNF-α’s biological activity is highly context- and time-dependent, reflecting a dynamic shift in receptor engagement during stroke progression. In the acute phase of ischemia, TNF-α predominantly signals through TNFR1, thereby promoting neurotoxic inflammation, apoptotic and necroptotic cell death, and disruption of the blood–brain barrier. In contrast, during the subacute and chronic phases, TNF-α increasingly exerts reparative and homeostatic effects via TNFR2, supporting cell survival, neuroprotection, angiogenesis, and tissue remodeling. This receptor-specific functional duality suggests that both excessive TNF-α production in the early phase and indiscriminate or prolonged blockade of TNF-α signaling may lead to unfavorable outcomes. Consequently, TNF-α serves not only as a sensitive indicator of inflammatory dynamics and clinical prognosis but also as a therapeutically challenging yet highly promising molecular target.

Conventional non-selective TNF inhibitors, although effective in systemic inflammatory disorders, are insufficient in the central nervous system because they suppress both the detrimental sTNF–TNFR1 signaling pathway and the beneficial, neuroprotective TNFR2-dependent signaling. As a result, current therapeutic approaches increasingly focus on temporally and receptor-selective modulation of TNF-α signaling. Selective sTNF inhibitors, such as XPro1595, and TNFR1-specific antagonists, including Atrosimab, have shown encouraging preclinical efficacy, particularly when administered during the early post-ischemic phase, when TNFR1-mediated mechanisms predominate.

Collectively, the available evidence suggests that the efficacy of future TNF-α-targeted therapies will critically depend on their ability to selectively inhibit pathogenic TNFR1-associated signaling during the acute phase of ischemic stroke, while preserving or enhancing TNFR2-mediated neuroprotective and regenerative processes during later stages. By integrating both temporal dynamics and receptor specificity, targeted modulation of TNF-α may mitigate early ischemic damage while simultaneously fostering long-term repair mechanisms, providing a promising conceptual basis for next-generation stroke therapies.

## Data Availability

No new data were created or analyzed in this study. Data sharing is not applicable to this article.

## Abbreviations

The following abbreviations are used in this manuscript:

BBB	blood–brain barrier
TNF-α	tumor necrosis factor alpha
NF-κB	nuclear factor kappa-light-chain-enhancer of activated B cells
ROS	reactive oxygen species
TNFR1; TNFRSF1A; CD120a; p55	tumor necrosis factor receptor 1
TNFR2; TNFRSF1B; CD120b; p75	tumor necrosis factor receptor 2
LPS	lipopolysaccharide
IL-1	interleukin 1
IL-6	interleukin 6
Munc13-4	mammalian uncoordinated-13 homolog 4
AP-1	activator protein-1
C/EBP	CCAAT/enhancer-binding protein
mTNF	transmembrane TNF
sTNF	soluble TNF
ADAM17	disintegrin and metalloproteinase domain-containing protein 17
TACE	TNF-α converting enzyme
DD	death domain
TRADD	TNF receptor-associated death domain protein
RIPK1/3	receptor-interacting protein kinase 1/3
TRAF2/5	TNF receptor–associated factors 2 and 5
cIAP1	cellular inhibitor of apoptosis protein-1
cIAP2	cellular inhibitor of apoptosis protein-2
LUBAC	linear ubiquitin chain assembly complex
IKK	IkappaB kinase
IKKα	inhibitor of nuclear factor κB kinase subunit alpha
IKKβ	inhibitor of nuclear factor κB kinase subunit beta
IκBα	Kappa light polypeptide gene enhancer in B-cells inhibitor alpha
IκBβ	Kappa light polypeptide gene enhancer in B-cells inhibitor beta
NEMO/IKKγ	nuclear factor κB essential modulator
MAP3K7	mitogen-activated protein kinase kinase kinase 7
TAK1	transforming growth factor beta–activated kinase 1
TAB1	TAK1-binding protein 1
TAB2	TAK1-binding protein 2
TAB3	TAK1-binding protein 3
JNK	c-Jun N-terminal kinase
p38 MAPK	p38 mitogen-activated protein kinase
FADD	Fas-associated death domain protein
FLIPL	FLICE-like inhibitory protein long isoform
MLKL	mixed lineage kinase domain like pseudokinase
CYLD	CYLD lysine 63 deubiquitinase
p50	NF-κB subunit p50
RelA	RELA proto-oncogene, NF-κB subunit p65
p100	kappa light polypeptide gene enhancer in B-cells inhibitor delta
NIK	NF-κB-inducing kinase
p52	NF-κB p52 subunit
RElB	RelB proto-oncogene, NF-κB subunit
TRAF2/3	TNF receptor–associated factors 2 and 3
ATP	adenosine triphosphate
RNS	reactive nitrogen species
NO	nitric oxide
CXCL1	neutrophil-recruiting chemokine
CCL2	monocyte-chemoattractant chemokine
MMPs	matrix metalloproteinases
DAMPs	damage-associated molecular patterns
HMGB1	high mobility group box 1
nNOS	neuronal NO synthase
eNOS	endothelial NO synthase
iNOS	inducible NO synthase
O_2_^•−^	superoxide
ONOO^−^	peroxynitrite
ZO-1	tight-junction protein
MPO	myeloperoxidase
gelatinase B/MMP-9	matrix metalloproteinase-9
gelatinase A/MMP-2	matrix metalloproteinase-2
stromelysin-1/MMP-3	matrix metalloproteinase-3
NLRP3	NOD-like receptor family, pyrin domain containing 3
IL-18	interleukin 18
IL-10	interleukin 10
IL-4	interleukin 4
TGF-β	transforming growth factor beta
T cell	T lymphocyte
TLR	toll-like receptor
MAPK	mitogen-activated protein kinase
CSF	cerebrospinal fluid
SSS	Scandinavian Stroke Scale
BI	Barthel Index
CSS	Canadian Stroke Scale
NIHSS	National Institutes of Health Stroke Scale
LACI	lacunar infarction
CT	computed tomography
MRI	magnetic resonance imaging
OND	other neurological diseases
TOAST	Trial of Org 10172 in Acute Stroke Treatment
SI	small-vessel infarction
LaI	large-artery infarction
mRS	modified Rankin Scale
POCI	posterior circulation infarction
CEI	cardioembolic infarct
GCS	Glasgow Coma Scale
GOS	Glasgow Outcome Scale
ODE	other determined etiology
I/PI	ipsilateral/peripheral infarction
LAAS	large artery atherosclerosis
NAT	neuroaxonal damage
CNS	central nervous system
EAE	experimental autoimmune encephalomyelitis
IgG1	immunoglobulin G1
NMDA	N-methyl-D-aspartate
